# Supplementary material for: Contribution of social determinants to symptoms of generalized anxiety disorder
Source: PLOS Ment Health. 2026 Mar 4;3(3):e0000552. doi: 10.1371/journal.pmen.0000552 (PMC12959655; doi:10.1371/journal.pmen.0000552)
Supplement: S1 Text — Percentage of respondents by age and biological sex. Table B. Percentage of respondents by country. Table C. Model results for all model types. Table D. Ranking of impact of factors on model performance (AUC and F1 scores) based on first or last inclusion in forward addition models. Table E. InfoGain values of each answer option for all ages. (DOCX) [file pmen.0000552.s001.docx]

Table A

| Demographic | % Respondents |
| --- | --- |
| 18-24 | 8.41% |
| 25-34 | 8.46% |
| 35-44 | 13.44% |
| 45-54 | 17.98% |
| 55-64 | 22.73% |
| 65-74 | 19.75% |
| 75-84 | 8.06% |
| 85+ | 1.18% |
| Male | 45.67% |
| Female | 53.74% |
| Other/Prefer not to say | 0.59% |

Table B

| Country | % of Respondents |
| --- | --- |
| United States | 17.20% |
| India | 11.27% |
| United Kingdom | 10.94% |
| Philippines | 9.83% |
| Kenya | 7.23% |
| South Africa | 6.73% |
| Nigeria | 6.24% |
| Pakistan | 5.62% |
| Australia | 3.64% |
| Trinidad and Tobago | 3.24% |
| Canada | 2.91% |
| Ireland | 2.08% |
| New Zealand | 1.96% |
| Sri Lanka | 1.89% |
| Singapore | 1.70% |
| Malaysia | 1.30% |
| Bangladesh | 1.16% |
| Uganda | 1.06% |
| Ghana | 1.02% |
| Zimbabwe | 0.80% |
| Other | 2.20% |

Table C

| **Performance for GAD-7 Score >10** | **AUC** | **Accuracy** | **Precision** | **Recall** | **F1** |
| --- | --- | --- | --- | --- | --- |
| **Logistic Regression** | **0.80** | **0.73** | **0.42** | **0.72** | **0.53** |
| Gradient Boosting | 0.75 | 0.79 | 0.52 | 0.25 | 0.33 |
| Random Forest | 0.79 | 0.79 | 0.50 | 0.47 | 0.49 |
| Naïve Bayes | 0.76 | 0.79 | 0.49 | 0.31 | 0.38 |
| **Performance for GAD-7 Score <10** |  |  |  |  |  |
| **Logistic Regression** | **0.80** | **0.73** | **0.91** | **0.73** | **0.81** |
| Gradient Boosting | 0.75 | 0.79 | 0.82 | 0.94 | 0.88 |
| Random Forest | 0.79 | 0.79 | 0.86 | 0.87 | 0.87 |
| Naïve Bayes | 0.76 | 0.79 | 0.83 | 0.91 | 0.87 |

Table D

|  |  |  | **AUC All Ages** | | | | **F1** | | | | **AUC 18-34** | | | | **F1 (18-34)** | | |  |
| --- | --- | --- | --- | --- | --- | --- | --- | --- | --- | --- | --- | --- | --- | --- | --- | --- | --- | --- |
|  | **Avg Rank all ages** | **Avg Rank 18-34** | **Added first** | **R** | **Added last** | R | **Added First** | **R** | **Added Last** | **R** | **Added first** | **R** | **Added last** | **R** | **Added First** | **R** | **Added Last** | **R** |
| Sleep | 1 | 1 | 0.163 | 1 | 0.041 | 1 | 0.125 | 2 | 0.034 | 1 | 0.082 | 1 | 0.026 | 1 | 0.150 | 1 | 0.013 | 1 |
| Employment | **3** | **6** | 0.108 | 2 | 0.017 | 2 | 0.072 | 6 | 0.007 | 3 | 0.026 | 7 | 0.006 | 6 | 0.004 | 7 | 0.006 | 5 |
| Social | 4 | 4 | 0.103 | 3 | 0.013 | 3 | 0.111 | 4 | -0.002 | 7 | 0.039 | 5 | 0.008 | 5 | 0.074 | 3 | 0.009 | 3 |
| Interpersonal Trauma | 3 | 2 | 0.086 | 4 | 0.007 | 4 | 0.195 | 1 | 0.007 | 2 | 0.071 | 2 | 0.013 | 2 | 0.116 | 2 | 0.010 | 2 |
| Exercise | 5 | 4 | 0.083 | 5 | 0.007 | 4 | 0.119 | 3 | -0.001 | 7 | 0.040 | 4 | 0.004 | 4 | 0.035 | 4 | 0.010 | 2 |
| Substance Use | 6 | 6 | 0.048 | 6 | 0.002 | 6 | 0.006 | 8 | 0.006 | 4 | 0.028 | 6 | 0.009 | 7 | -0.022 | 8 | 0.008 | 4 |
| Education | 6 | 8 | 0.043 | 7 | 0.002 | 5 | 0.073 | 5 | 0.000 | 6 | -0.028 | 9 | -0.014 | 9 | 0.019 | 6 | -0.008 | 6 |
| Financial Adversities | 7 | 8 | 0.036 | 8 | -0.001 | 6 | 0.032 | 7 | 0.005 | 5 | 0.009 | 8 | -0.003 | 8 | -0.009 | 9 | -0.007 | 6 |
| Other Adversities | **7** | **4** | 0.036 | 9 | -0.001 | 6 | 0.032 | 7 | 0.005 | 5 | 0.045 | 3 | -0.008 | 3 | 0.023 | 5 | -0.007 | 6 |

Table E

| # | Feature | InfoGain |
| --- | --- | --- |
| 1 | Frequency of getting a good nights sleep | 0.074 |
| 2 | Frequency of getting a good nights sleep=Hardly ever | 0.042 |
| 3 | Frequency of getting a good nights sleep=Most of the time | 0.032 |
| 4 | Frequency of Socializing=Rarely/Never | 0.031 |
| 5 | Frequency of doing exercise=Rarely/Never | 0.025 |
| 6 | Employment=Retired | 0.022 |
| 7 | Frequency of Socializing=1 a week | 0.018 |
| 8 | Prolonged or sustained bullying in person from peers | 0.015 |
| 9 | Prolonged emotional or psychological abuse or neglect from parent/caregiver | 0.014 |
| 10 | Education | 0.013 |
| 11 | Frequency of getting a good nights sleep=All of the time | 0.013 |
| 12 | Employment=Not able to work | 0.012 |
| 13 | Frequency of getting a good nights sleep=Some of the time | 0.010 |
| 14 | Employment=Unemployed | 0.009 |
| 15 | Prolonged physical abuse\| or severe physical assault CT | 0.009 |
| 16 | Threatening\| coercive or controlling behavior by another person | 0.008 |
| 17 | Physical violence in the home between family members | 0.007 |
| 18 | Sedatives or Sleeping Pills | 0.007 |
| 19 | Education=High School | 0.006 |
| 20 | Threatening\| coercive or controlling behavior by another person CT | 0.006 |
| 21 | Frequency of doing exercise=Every day | 0.006 |
| 22 | Frequency of doing exercise=Few days a week | 0.006 |
| 23 | Cyberbullying or online abuse | 0.005 |
| 24 | Loss of your job or livelihood leading to an inability to make ends meet. | 0.005 |
| 25 | Tobacco products | 0.005 |
| 26 | Extreme poverty leading to homelessness and/or hunger. | 0.005 |
| 27 | None of the above | 0.004 |
| 28 | Employment=Homemaker | 0.004 |
| 29 | Employment=Studying | 0.004 |
| 30 | Prolonged sexual abuse\| or severe sexual assault. | 0.003 |
| 31 | Vaping products | 0.003 |
| 32 | Education=Master's Degree | 0.002 |
| 33 | Opioids | 0.002 |
| 34 | Lived with a parent/caregiver who was an alcoholic or who regularly used street drugs | 0.002 |
| 35 | Life threatening or debilitating injury or illness. | 0.002 |
| 36 | Education=Bachelor's Degree | 0.002 |
| 37 | Parental Divorce or family breakup | 0.002 |
| 38 | Biological Sex=Prefer not to say | 0.002 |
| 39 | Education=Some High School | 0.001 |
| 40 | Frequency of Socializing=Once a week | 0.001 |
| 41 | Education=Associate‚Äö√Ñ√¥s Degree | 0.001 |
| 42 | Displacement from your home due to political\| environmental or economic reasons CT | 0.001 |
| 43 | Cannabis | 0.001 |
| 44 | Employment=Employed /Self employed | 0.001 |
| 45 | Caring for a child or partner with a major chronic disability or illness | 0.001 |
| 46 | Biological Sex=Other/Intersex | 0.001 |
| 47 | Displacement from your home due to political\| environmental or economic reasons | 0.001 |
| 48 | Education=M.D. | 0.001 |
| 49 | Education=PhD | 0.001 |
| 50 | Sudden or premature death of a loved one | 0.001 |
| 51 | Sudden or premature death of a parent or sibling | 0.000 |
| 52 | Education=Prefer not to say | 0.000 |
| 53 | Frequency of doing exercise=Once a week | 0.000 |
| 54 | Suffered a loss in a major fire\| flood\| earthquake\| or natural disaster | 0.000 |
| 55 | Suffered a loss in a major fire\| flood\| earthquake\| or natural disaster CT | 0.000 |
| 56 | Frequency of doing exercise=Less than once a week | 0.000 |
| 57 | Education=Other | 0.000 |
| 58 | Alcoholic beverages | 0.000 |
| 59 | Education=Primary Education | 0.000 |
| 60 | Frequency of Socializing=1-3 times a month | 0.000 |
| 61 | Divorce/separation or family breakup | 0.000 |
| 62 | Involvement or close witness to a war | 0.000 |
| 63 | Education=Vocational certification | 0.000 |
| 64 | Education=J.D. | 0.000 |
| 65 | Amphetamine type stimulants (e.g. speed\| diet pills\| ecstasy\| etc.) | 0.000 |
| 66 | Forced family control over major life decisions (e.g. marriage) | 0.000 |
| 67 | Forced family control over major life decisions(Childhood) | 0.000 |
| 68 | Life threatening or debilitating injury or illness (Childhood) | 0.000 |
| 69 | I did not experience any of the above | 0.000 |
