# Supplementary material for: Contribution of social determinants to symptoms of generalized anxiety disorder
Source: PLOS Ment Health. 2026 Mar 4;3(3):e0000552. doi: 10.1371/journal.pmen.0000552 (PMC12959655; doi:10.1371/journal.pmen.0000552)
Supplement: Attachment — Submitted filename: Response to Reviewers.docx [file pmen.0000552.s003.docx]

January 13, 2026

Karli Montague-Cardoso

Staff Editor

PLOS Mental Health

**Re:** Revised Manuscript - Contribution of Social Determinants to Symptoms of Generalized Anxiety Disorder

Dear Dr. Montague-Cardoso,

Thank you for the opportunity to revise our manuscript. We appreciate the constructive feedback from reviewers and the editorial office, which has strengthened our paper. Below, we address the editorial concerns about text overlap, followed by responses to each reviewer’s comment.

# Response to Editorial Comment on Text Overlap

We thank the editor for noting the text similarity with our previous publication (Bala et al., BMJ Open 2024; doi:10.1136/bmjopen-2023-075095). We wish to clarify that while both studies use data from the Global Mind Project and employ similar machine learning methodology, they address distinct research questions and outcomes:

The BMJ Open paper examines predictors of transdiagnostic mental health status using the Mental Health Quotient (MHQ), an aggregate measure spanning 47 symptoms across 10 disorders, in a sample of 270,000 individuals collected between April 2020 and December 2021.

The current manuscript examines predictors of anxiety-specific symptoms using the GAD-7, a validated clinical screening tool for Generalized Anxiety Disorder, in a distinct and smaller sample of 4,186 individuals collected in September 2022.

The key scientific contributions that distinguish this work include:

1. Application to a disorder-specific outcome (GAD-7) rather than transdiagnostic distress, allowing comparison of anxiety-specific versus general mental health predictors.

2. Identification that sleep quality plays an even more dominant role in anxiety symptoms than in overall mental distress (ranked 1st for GAD-7 vs. 2nd-3rd for MHQ).

3. Different hierarchy of factors: social interaction was the top contributor for MHQ prediction, whereas sleep quality dominated for GAD-7 prediction.

4. Use of forward-selection methodology to assess factor contributions both inclusive of and independent of correlations, complementing the SHAP and Information Gain approaches.

We have revised the manuscript to ensure all methodological descriptions that necessarily overlap (e.g., Global Mind Project recruitment procedures, data quality criteria) are appropriately cited to the original BMJ Open source. We have also expanded the Discussion section ("Similarity of factors to transdiagnostic predictions") to explicitly compare findings between the two studies, noting both consistencies and important differences.

Regarding the second flagged reference (Chen et al., 2025; doi:10.3389/fpsyt . 2025.1576964), this is an independent study by researchers at Shanghai Normal University examining anxiety-depression symptom networks in older Chinese adults living alone using data from the China Longitudinal Healthy Longevity Survey (CLHLS). This paper is entirely unrelated to our research group and uses a different dataset, a different population, and a different analytical approach (network analysis vs. machine learning classification). Any textual similarity is likely attributable to: (1) both studies using the GAD-7 instrument and citing the same validation reference (Spitzer et al., 2006); (2) standard methodological terminology common to cross-sectional mental health research; and (3) both studies employing LASSO regularization, which requires similar technical descriptions. We have reviewed our manuscript carefully and confirm that there is no substantive text overlap with this independent work beyond these standard elements. No changes to our manuscript are required with respect to this reference.

# Response to Reviewer #1

**Comment 1 (Abstract clarity):** *The reviewer noted that the abstract gives the impression of a hodge-podge of predictor types and lacks information on sample size, design, or strength of associations.*

**Response:** We have revised the abstract to clarify that the aim is to evaluate aggregate predictive associations rather than causal pathways. We have also ensured the sample size (N=4,186) and cross-sectional design are clearly stated, along with key performance metrics.

**Comment 2 (Line 52 - GAD prevalence):** *The reviewer correctly noted that specific phobia and adjustment disorder are more common than GAD.*

**Response:** We have revised the sentence to state that GAD is "among the most prevalent and clinically significant anxiety-related conditions" rather than claiming it is the most common.

**Comment 3 (Line 89 - Sentence structure):** *The reviewer identified a grammatical error.*

**Response:** We have corrected the sentence to read "as well as collecting responses on a rich array of lifestyle and life experience factors."

**Comment 4 (Line 95 - "Predicting" terminology):** *The reviewer noted that "predicting" is inappropriate for a cross-sectional study with bidirectional associations.*

**Response:** We agree and have revised the text to clarify that our analysis examines statistical classification of symptom severity rather than prospective prediction. This distinction is now made explicit in both the abstract and introduction.

**Comment 5 (Lines 122-123 - SD exclusion rationale):** *The reviewer requested clarification of the SD > 0.2 exclusion criterion.*

**Response:** We have expanded this section to explain that this criterion is part of the validated MHQ quality-control framework, where SD < 0.2 across 47 items indicates extremely low response variability (e.g., selecting the same response for nearly all items), a pattern empirically associated with disengaged or inattentive responding. This criterion is consistent with our previous work [59].

**Comment 6 (Line 132 - Sample calibration):** *The reviewer inquired about whether calibration was used to make the sample representative.*

**Response:** We have added a statement clarifying that no post-stratification weights were applied, as the study's primary purpose was model performance evaluation rather than population prevalence estimation.

**Comment 7 (Line 150 - Two models rationale):** *The reviewer questioned why separate models for GAD ≥10 and <10 were estimated.*

**Response:** We have clarified that this approach trains separate classifiers on minority and majority classes to mitigate imbalanced-data bias, and that this is an overfitting-mitigation strategy rather than a method for imputing missing outcome data.

**Comment 8 (Analysis approach, LASSO benchmark, and Table 4 clarity):** *The reviewer noted that they would expect LASSO as a benchmark model and that Table 4 does not explain the meaning of "impact" or which classifier the results are based on.*

**Response:** We thank the reviewer for this important clarification. We wish to note that our Logistic Regression model was indeed implemented with L1 (LASSO) regularization using the Orange Data Mining platform (C=12, with class balancing enabled). We have revised the Methods section to explicitly state this. Supplementary Table 3 provides the full model comparison showing that LASSO-regularized Logistic Regression achieved the best AUC (0.80) and most balanced performance across classes compared to Gradient Boosting, Random Forest, and Naïve Bayes.

Regarding Table 4, we have expanded the legend to clarify that "impact" refers to the change in AUC or F1 score when each variable group is added in the forward-selection framework using the LASSO-regularized Logistic Regression classifier. The forward-selection approach (adding variables first vs. last) provides complementary insight to SHAP: adding first captures contribution inclusive of correlations with other factors, while adding last reveals independent contribution after accounting for shared variance. The actual delta values are provided in Supplementary Table 4.

**Comment 9 (SHAP interpretation):** *The reviewer cautioned that SHAP values do not unequivocally indicate predictor importance when predictors are correlated, and that our interpretations are naïve.*

**Response:** We appreciate this important methodological point. We have revised the SHAP section to frame it as a qualitative tool for highlighting directionality and consistency rather than definitive causal importance and added a note explaining that correlated predictors share importance estimates because SHAP values summarize contributions across all feature combination scenarios.

Importantly, we used SHAP as one of multiple complementary methods. The forward-selection analysis (Table 4, Supplementary Table 4) directly addresses the collinearity concern by showing variable contributions both with (added first) and without (added last) correlations. The consistency of findings across Information Gain, forward-selection, and SHAP—with sleep quality ranking highest across all methods—strengthens confidence in the conclusions despite the inherent limitations of any single approach.

# Response to Reviewer #2

**Comment 1 (Social determinants terminology):** *The reviewer noted that the term "social determinants" appears in the title but not in the manuscript.*

**Response:** We note that the term does appear in the Introduction, where we state that "people's environment, life experiences, and lifestyle habits (together, social determinants) all play a prominent role." We believe this adequately defines the term in context and aligns with our previous work on this topic [59].

**Comment 2 (Variable operationalization):** *The reviewer requested more information on how variables were measured.*

**Response:** We have added a sentence in the Methods section clarifying that all life context factors were assessed via self-report items with categorical response options (e.g., frequency scales for lifestyle factors, binary presence/absence for adversities, and multiple-choice options for demographics). Full details of the assessment instrument are available in our previous methodological publications [51, 52].

**Comment 3 (Inclusion criteria):** *The reviewer requested clarification of the inclusion criteria.*

**Response:** This has been addressed as described in our response to Reviewer 1, Comment 5 above.

**Comment 4 (Imbalanced datasets and LR performance):** *The reviewer requested an explanation of "imbalanced datasets" and why LR didn't achieve the highest scores.*

**Response:** We have clarified that "imbalanced" refers to unequal class distributions (only 24% had GAD-7 ≥10). We have also explained that LR's balanced performance results from tree-based models maximizing overall accuracy at the expense of minority-class detection, whereas LR provides more stable probability estimates across classes. We have also clarified that "geographies" refers to Western/developed and non-Western/developing countries and added a statement on listwise deletion for missing data.

**Comment 5 (Age finding interpretation):** *The reviewer found the age-related findings counterintuitive.*

**Response:** We have expanded the Discussion to explain that while adverse events are commonly reported among younger populations, the specific constellation of factors captured here—many representing cumulative life experiences—may be more salient risk markers for older adults, whereas younger adults may be more affected by contemporary factors not included in the model (e.g., social media use, diet quality). This pattern is consistent with our findings from the MHQ study [59], where model performance also improved systematically with age.

**Comment 6 (Limitations):** *The reviewer suggested noting the limitations of self-reported measures and ML techniques.*

**Response:** We have added text to the Limitations section acknowledging that all measures were self-reported (subject to recall bias and social desirability effects) and that ML techniques, while advantageous for handling complex relationships, are susceptible to overfitting and may not generalize to populations with different characteristics.

We believe these revisions have substantially strengthened the manuscript and adequately addressed all editorial and reviewer concerns. We thank the reviewers for their thoughtful and constructive feedback.

Sincerely,

Tara Thiagarajan
